# Changing trend? Sex ratios of children born to Indian immigrants in Norway revisited

**DOI:** 10.1186/1471-2393-13-170

**Published:** 2013-09-05

**Authors:** Marianne Tønnessen, Vebjørn Aalandslid, Terje Skjerpen

**Affiliations:** 1 Statistics Norway, PO Box 8131 Dept, NO-0033 Oslo, Norway

## Abstract

**Background:**

In some Western countries, a disturbingly low share of girls has been observed among new-borns from Indian immigrants. Also in Norway, a previous study based on figures from 1969–2005 showed a high percentage of boys among children of Indian origin living in Norway, when the birth was of higher order (third birth or later). This was suggested to reflect a practice of sex-selective abortions in the Indian immigrant population. In this article we have seen whether extended time series for the period 2006–2012 give further support to this claim.

**Methods:**

Based on data from the Norwegian Central Population Register we used observations for the sex of all live births in Norway for the period 1969–2012 where the mother was born in India. The percentage of boys was calculated for each birth order, during four sub periods. Utilising a binomial probability model we tested whether the observed sex differences among Indian-born women were significantly different from sex differences among all births.

**Results:**

Contrary to findings from earlier periods and other Western countries, we found that Indian-born women in Norway gave birth to more girls than boys of higher order in the period 2006–2012. This is somewhat surprising, since sex selection is usually expected to be stronger if the mother already has two or more children.

**Conclusions:**

The extended time series do not suggest a prevalence of sex selective abortions among Indian-born women in Norway. We discuss whether the change from a majority of boys to a majority of girls in higher order could be explained by new waves of immigrant women, by new preferences among long-residing immigrant women in Norway – or by mere coincidence.

## Background

A number of studies have discussed whether an observed low share of girls among some immigrant groups in Western Europe and North America can be attributed to a practice of sex selective abortions [1-5]. In some regions of Asia, and especially in India where son preference is strong, the female to male ratio is disturbingly low. Sex selective abortions may, if conducted on a large scale, disturb the sex balance of a society. It is also a sign of an attitude towards women that is condemned in modern societies.

The last Indian population census showed that among children 0–6 years old the number of girls per 1000 boys decreased from 941 in 1991 to 913 in 2011 [6], corresponding to an increase in the percentage of boys from 51,52 to 52,27 per cent. Together with female infanticide, the skewed sex ratio is believed to be caused by sex selective abortions [7]. The share of girls is often found to be especially low among higher birth orders in families without male offspring [7], confirming a hypothesis that families do not take action to make sure their next child is a boy until they have already got some children, but no or few sons.

Singh *et al*. [8] investigated whether the same low female to male ratios could be found among Indian and Pakistani immigrants living in Norway, indicating a practice of sex selective abortions in a Norwegian context. They studied live births of mothers of Indian and Pakistani origin for the period 1969–2005 and calculated the female to male sex ratios, in increasing birth order, for three periods (1969–1986, 1987–1996 and 1997–2005). The study found significant imbalances in the sex ratio of children born to mothers of Indian origin, but only from the mid 1980s and onwards and only for the third and fourth birth order. The tendency was not observed among births of children of Pakistani origin. The authors linked the decline in female births to the use of ultrasound scanning technology, introduced in Norway in 1987, and suggested that the imbalance, in the third and fourth birth order, could stem from sex selective abortions due to prenatal sex determination by ultrasound.

In Norway, the general abortion limit is at the 12th week of pregnancy. Ultrasound techniques that may reveal the foetus’ sex is normally given around week 17–19. To have an induced abortion after week 12, one has to apply to a medical committee which does not accept sex selection as a valid reason for abortion. Hence, sex selective abortions after ultrasound would have to be performed abroad.

The aim of this study is to see whether extended time series for the period 2006–2012 give further support to the hypothesis of sex-selective abortions among Indian immigrants to Norway.

## Methods

*Sex ratio* is the ratio of males to females in a given population, often expressed as the number of males for every 100 females. In this study, we use *percentage of boys* as a complementary measure.

### Data collection and descriptive statistics

The micro-data used in this study is based on administrative data from the Central Population Register, managed by Statistics Norway under the Norwegian Statistics Act.^a^ The data include all live births in Norway for the years 1969–2012. Of these, 4,619 births were registered as births given by Indian-born mothers. Information about birth order was derived from the same source. To determine the birth parity, siblings registered in Norway, but born abroad (by the same mother) were also included.

Compared to the study by Singh *et al*., we also present results from an additional time period – 2006–2012 (added to the three periods considered by Singh *et al*.: 1969–1986, 1987–1996 and 1997–2005). In total, we studied 4,619 live births of Indian-born mothers, 857 in the first period and 1,204, 1,186 and 1,372 in the 2nd, 3rd and 4th period, respectively. Of these, 2,156 were first born, 1,690 second born, 587 third born, 135 fourth born and 51 were of higher than fourth birth order. Of the children born as number three or four, 37 and 29 per cent respectively, had only female siblings.

In studies of small groups, small changes in the definitions of who to include or exclude will have consequences for the overall number of observations. If we only observed births where the mother was Indian-born, and where she had no Norwegian-born parents or grandparents, the overall number of observations would decrease from 4,619 to 4,117. If we also excluded the births where the father was Norwegian-born, the number of observations would be further reduced to 3,636. However, as was done in the study of Singh *et al*. we only observed the birth patterns of Indian-born mothers, and have chosen not to include information about the father in our study.

### Statistical analysis

To test whether the sex ratios among the Norwegian-Indian births were significantly different from the average sex ratio of all Norwegian births we used a binomial probability model analysing each period and each birth order separately. From the total population, the calculated probability (p_0_) of giving birth to a boy is 0.5136. The corresponding probability of a girl is q_0_ = 1-p_0_. Let X be a stochastic variable representing the number of male births among all births of Indian-born mothers corresponding to a given period and a given birth order. We assume that X is binomially distributed with parameters N (number of births corresponding to period and birth order) and p (the probability parameter). Since there is no reason to expect selective abortions in disfavour of boys, we found it appropriate to consider a one-sided hypothesis:

$$H_{0}:p=p_{0}\mathrm{vs}.\;H_{1}:p>p_{0}.$$

A high value of X would indicate that H_0_ is false and that a high prevalence of boys is not based on coincidence. Since we in all cases have min(Np_0_, Nq_0_) >10, the statistical inference may be based on the normal distribution [9]. The estimator of p is given by $\hat{p}=\frac{X}{N}$, and the standard error is given by $\mathrm{Std}\left(\frac{X}{N}\right)=\sqrt{\frac{\mathrm{pq}}{N}}.$ If H_0_ is true, $Z=\frac{\hat{p}-p_{0}}{\sqrt{\frac{p_{0}q_{0}}{N}}}$ will be approximately standard normally distributed. The realized value of Z, which we label z, can be compared to the critical value determined by the chosen significance level. At the 10 per cent significance level the critical value is 1.2815, whereas it is 1.645 and 2.325 at the 5 and 1 per cent significance levels, respectively.

## Results

**Figure 1:**
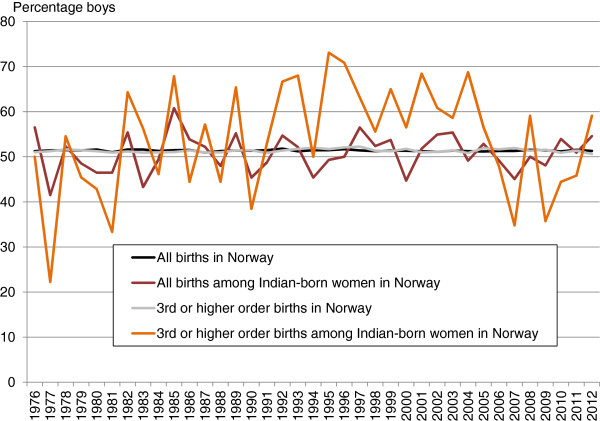
**Percentage of boys.** Percentage of boys born in Norway to Indian-born women, compared with all births in Norway, 1976–2012.

In Figure 1, the percentage of boys born to Indian-born women in Norway is compared to the percentage of boys among all births in Norway in the period 1976–2012. A separate line is also shown for the percentage of boys in third or higher order births among Indian-born mothers, which may be compared to the percentage of boys among all third or higher order births in Norway.

The figure shows two main trends: First, the percentage of boys among the new-borns from Indian-born mothers in Norway fluctuates a lot from year to year, and particularly for the higher order births (sibling number three or more). This is not surprising as the number of observations is relatively low (the total number of yearly births among Indian-born women in Norway was below 100 until 1988 and below 200 until 2009 – whereas the yearly number of third or higher order births was less than 20 before 1985, and has since stayed between 16 and 28).

Second, although changes from one year to another may be just a result of chance, we see that in the period between around 1990 and 2005, the Indian-born women in Norway gave birth to more boys than girls in higher birth order. However, after 2005, the trend seems to have changed: As a whole, more girls than boys have been born in these birth orders in this last period.

**Figure 2:**
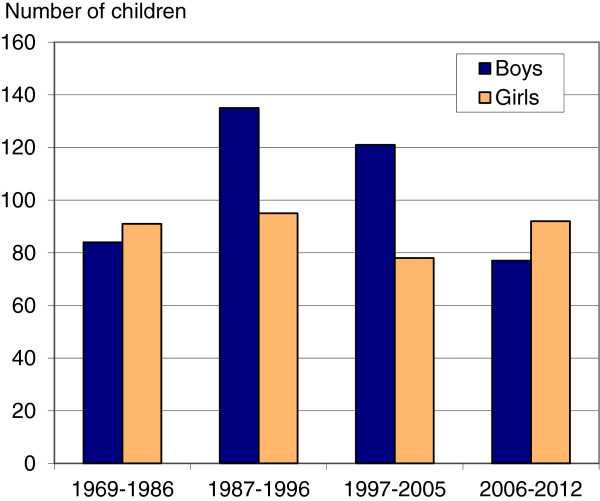
**Number of boys and girls in higher birth order.** Number of boys and girls in third and higher birth order born in Norway to Indian-born mothers, 1969–2012.

The trend change among higher birth orders is also shown in Figure 2 – with absolute numbers of births for the four time periods. From the second period on, i.e., from the time when ultrasound technology was made generally available in Norway, a large majority of boys were born in these higher birth orders. However, the last period shows an opposite tendency – in spite of available ultrasound technology and relatively cheap international airline tickets.

Are the variations shown only a matter of coincidences? Our binominal probability analysis (Table 1) reveals three main findings:

**Table 1:** Binomial probability analysis of births by Indian-born women living in Norway, by sex and birth order, 1969-2012

	**Period**			
	1969-1986	1987-1996	1997-2005	2006-2012
*First order births*				
Male births/all births	187/378	259/543	258/533	357/702
Percentage boys	49.5	47.7	48.4	50.9
z	−0.735	−1.707	−1.365	−0.268
*Second order births*				
Male births/all births	157/304	209/431	243/454	259/501
Percentage boys	51.6	48.5	53.5	51.7
z	0.099	−1.191	0.923	0.151
*Third order births*				
Male births/all births	55/117	98/170	99/167	57/133
Percentage boys	47.0	57.6	59.3	42.9
z	−0.942	1.640*	2.048**	−1.962
*Fourth order births*				
Male births/all births	19/35	30/46	17/24	16/30
Percentage boys	54.3	65.2	70.8	53.3
z	0.346	1.880**	1.909**	0.216

*: Significant at the 0.10 level.

**: Significant at the 0.05 level.

Since we consider testing of one-sided hypotheses, we do not report (two-sided) confidence intervals for the probability parameter.

For fifth or higher birth orders, observations were few and no significant discrepancies were found.

*First*, no significant discrepancies from the average percentage of boys were found for first and second order births.

*Second*, for third and fourth order births, in the two first periods after ultrasound technology was made available (1987–1996 and 1997–2005), the percentages of boys among new-borns were significantly higher than normal (significant at the 0.1 or 0.05 level). However, the discrepancies were not large enough to be significant at the 0.01 level. The risk of getting such a high share of boys without intervention, can thus be interpreted to lie between 1 and 5 per cent (or in one case, 10 per cent).

*Third*, for the last period (2006–2012) there were no significant results indicating any sex selection in disfavour of girls in any of the birth orders. For third order, only 42.9 per cent of the children were boys (in fourth order there was still a male majority, 16 boys and 14 girls). Since our test is one-sided, we did not test for discrepancies in the other direction (i.e., we did not look for sex selection in the disfavour of boys). A two-sided test however (with its critical value of z=1.96 at the 5 per cent level), would have revealed that the percentage of boys in third order births was significantly (at the 0.05 level) different from P_0_=0.5136.

Our two first findings are in line with results from previous empirical analyses and theories about sex discrimination not appearing until for siblings of higher orders [3,4,7]. The observations from the last period, however, deserve some more elaboration.

**Figure 3:**
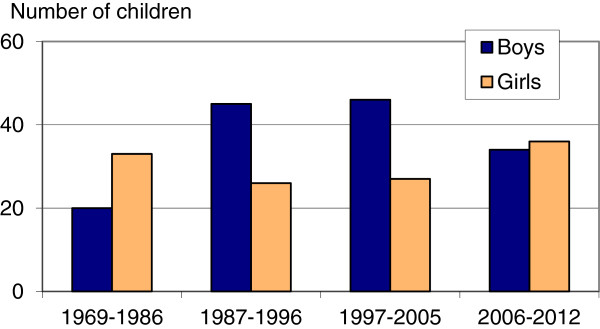
**Number of boys and girls where all previous births are girls.** Number of boys and girls in third and higher birth order born in Norway to Indian-born women who previously had only girls, 1969–2012.

As already mentioned, other studies have shown that the discriminatory practices are particular prevalent in families with only girls. We examined the high order sex ratios for families where all the previously born children are female, to see if the trend had changed for them as well. As shown in Figure 3, even in this group, where sex selective abortion would be most expected, the last period showed a small majority of girls being born. Results from the significance test (Table 2) show a similar trend as for all third and higher order births among Indian-born mothers: Some (slightly significant) discrepancies from a normal sex distribution for third order births in the two first periods after 1986, but no signs of sex selection in the last period. We disregarded fourth and higher order births for this group because of the low number of observations.

**Table 2:** Binomial probability analysis of births by Indian-born women living in Norway where all older siblings are girls, by sex and birth order, 1969-2012

	**Period**			
	1969-1986	1987-1996	1997-2005	2006-2012
*Second order births*				
Male births/all births	76/152	110/224	122/240	124/259
Percentage boys	50.0	49.1	50.8	47.9
z	−0.335	−0.675	−0.163	−1.121
*Third order births*				
Male births/all births	14/39	35/57	40/65	27/57
Percentage boys	35.9	61.4	61.5	47.4
z	−1.932	1.517^*^	1.642^*^	−0.603

*: Significant at the 0.10 level.

Since we consider testing of one-sided hypotheses, we do not report (two-sided) confidence intervals for the probability parameter.

## Discussion

Why did the trend change? It could have been due to new Indian immigrants entering Norway. The Indian-born immigrant population residing in Norway increased from 4,600 in 2005 to 8,700 in 2012. However, Indian-born women who migrated to Norway after 2005 gave birth to less than 10 children in third or higher order (with a majority of boys) in the last period. It is among Indian-born women who have resided more than 6 years in Norway that we observed a majority of female births in the third and fourth order (90 girls vs 72 boys in the last period). Hence, the change in the observed sex ratio did not originate from new immigration from India.

Does this mean that the preferences for sons have changed among the Indian-born mothers who have resided many years in the relatively gender-equal Norwegian society? To find indications of son preference, we examined whether mothers who had only girls, were overrepresented among those who chose to have another child. If mothers with two girls often choose to have another child, while mothers with two boys (or with one child of each sex) do not, this may be an indication of preference for sons.

Among all third or higher order children born to any mother in Norway in the period 2006–2012, 24 per cent had only brothers, whereas 21 per cent had only sisters. Among the children born to Indian-born mothers the corresponding shares were 15 and 41 per cent – meaning that fewer had only brothers and a considerably higher share had only sisters. Thus, Indian-born mothers more often got another child if they had only daughters compared to if they had only sons. Although the general trend among Indian-born mothers in Norway has been that fewer choose to have more than two children, the share of Norwegian-Indian new-borns in higher birth orders who have only female siblings actually increased over the time periods, which indicates a persisting preference for sons among some Indian-born mothers in Norway. However, as our extended data material show: this preference does not seem to be translated into sex selective abortions.

Almond *et al*. [4] point out that sex selection and continued childbearing are alternative ways of achieving a son, and that there may be some substitution between the two strategies; lower fertility levels may increase the use of sex selective abortions and vice versa. They found signs among South and East Asian immigrants in Canada of a relative substitution (across generations) towards the abortion route from the fertility route. Our results may suggest some substitution over time in the opposite direction.

The suggested interpretation above does not, however, explain why we have seen a *majority* of girls among third order births in the last period. Although the percentage of boys in third order is so low that it would be significant at the 0.05 level if we had used a two-sided test, neither previous theory nor our analyses of preferences suggest that the surplus of girls has been anything but a matter of coincidence. As shown in Figure 1, there are large fluctuations in these numbers from year to year. Consequently, if this last surplus was due to mere coincidence, the surplus of boys in earlier periods could just as well be due to random deflections.

## Conclusions

Extended time series on percentage of boys among children born to Indian immigrants in Norway do not suggest a practice of sex selective abortions. While a previous study [8] found a significantly skewed sex ratio among higher order births after ultrasound was introduced in Norway, updated figures from 2006–2012 indicate that the trend has changed: As a whole, Indian-born women in Norway have given birth to more girls than boys in higher birth orders in this last period. The number of births is however low, and the annual variations are therefore large, making it difficult to draw clear-cut conclusions from the data.

## Endnote

^a^The project is also approved by the Data Protection Official for Research (personvernombudet) as prescribed by the routines of Statistics Norway in accordance with the Person Information Act (personopplysningsforskriften § 7–12). The Norwegian Statistics Act precludes us from making the data publicly available, but Statistics Norway can provide access subject to approval of an application. Documentation that sufficient confidentiality can be guaranteed, as well as the consent of the Norwegian Data Inspectorate, may be required.

## Competing interests

The authors declare that they have no competing interests.

## Authors’ contributions

All the authors contributed to the study design and drafted different parts of the manuscript. MT carried out the data analyses, VAA participated in literature review, and TS performed the binominal probability analyses. All authors have read and approved the final manuscript.

## Pre-publication history

The pre-publication history for this paper can be accessed here:

http://www.biomedcentral.com/1471-2393/13/170/prepub
